# Mitochondrial calcium uniporter as a target of microRNA-340 and promoter of metastasis via enhancing the Warburg effect

**DOI:** 10.18632/oncotarget.19747

**Published:** 2017-07-31

**Authors:** Changhui Yu, Yuhao Wang, Jiawen Peng, Qiang Shen, Mimi Chen, Wei Tang, Xiumei Li, Chunqing Cai, Bin Wang, Shaoxi Cai, Xiaojing Meng, Fei Zou

**Affiliations:** ^1^ Department of Occupational Health and Occupational Medicine, Guangdong Provincial Key Laboratory of Tropical Disease Research, School of Public Health, Southern Medical University, Guangzhou, China; ^2^ Department of Respiratory and Critical Care Medicine, Chronic Airways Diseases Laboratory, Nanfang Hospital, Southern Medical University, Guangzhou, China; ^3^ Department of Clinical Cancer Prevention, The University of Texas MD Anderson Cancer Center, Houston, Texas, U.S.A; ^4^ Guangdong Provincial Key Laboratory of Gastroenterology, Department of Gastroenterology, Nanfang Hospital, Southern Medical University, Guangzhou, China

**Keywords:** breast cancer, metastasis, Warburg effect, mitochondrial calcium uniporter, microRNA-340

## Abstract

**Background:**

A shift from oxygen phosphorylation to aerobic glycolysis was known as the Warburg effect and a characteristic of cancer cell metabolism facilitating metastasis. Mitochondrial calcium uniporter (MCU), a key ion channel that mediates Ca^2+^ uptake into mitochondria, was found to promote cancer progression and metastasis. However, its explicit role in shifting metabolism of breast cancer cells has not been defined.

**Methods:**

We evaluated MCU overexpression or knock-down on migration, invasion and glucose metabolismin breast cancer cells. Mitochondrial Ca^2+^ dynamics were monitored with Rhod-2 fluorescence imaging. Luciferase reporter assay was used to confirm the interaction between miR-340 and 3’-untranslated region (3’-UTR) of *MCU* gene. Mouse models of lung metastasis were used to determine whether gain-/loss-of-MCU impacts metastasis. MCU expression was assessed in 60 tumor samples from breast cancer patients by immunohistochemistry (IHC).

**Results:**

Knockdown of MCU in MDA-MB-231 cells significantly reduced cell migration and invasion *in vitro* and lung metastasis *in vivo*; whereas overexpression of MCU in MCF-7 cells significantly increased migration and invasion *in vitro* and lung metastasis *in vivo*. Overexpression of MCU promoted lung metastasis by enhancing glycolysis, whereas suppression of MCU abolished this effect. Moreover, a novel mechanism was identified that MCU was a direct target of microRNA-340, which suppressed breast cancer cell motility by inhibiting glycolysis. Consistently, significantly increased MCU protein was found in metastatic breast cancer patients.

**Conclusions:**

We identified a novel mechanism that upregulated MCU promotes breast cancer metastasis via enhancing glycolysis, and that this process is posttranscriptionally and negatively regulated by microRNA-340.

## INTRODUCTION

The metabolic shift known as the Warburg effect is a well-recognized hallmark of cancer cells. Cancer cells rely more on aerobic glycolysis than on mitochondrial oxidation for energy production, even in the presence of normal oxygen levels [1, 2]. This enhanced glycolysis induces glucose intake, production less adenosine triphosphate (ATP) and insufficient intermediate metabolites (such as reduced nicotinamide adenine dinucleotide; acetyl coenzyme A; and ribose), and an increase in lactate production to meet the energy needs of cancer cells for growth and metastasis [1–4]. In particular, increased lactate production generates an acidic environment that facilitates cancer cell motility, eventually leads to the development of metastasis [5].

Increasing evidence demonstrates that mitochondria also play a vital role in metabolic regulation in cancer cells [6–11]. Previously, we reported that mitochondrial calcium uniporter (MCU) increases mitochondrial Ca^2+^ entry and ROS to impact cancer cell motility [12, 13]. Recently, MCU was found to act as a major Ca^2+^ channel in the mitochondrial inner membrane and plays a key role in mitochondrial Ca^2+^ ([Ca^2+^]_m_) uptake and mitochondrial function [14, 15]. Furthermore, MCU expression correlates with mammary tumor size and lymph node infiltration in mice and is an indicator of poor prognosis for breast cancer [16]. Silencing of MCU markedly decreases [Ca^2+^]_m_ accumulation, metastatic cell motility, and matrix invasiveness in breast cancer cells [17]. However, an explicit role of MCU in regulating glycolysis in breast cancer cells has yet to be determined.

The microRNAs (miRNAs) are noncoding small RNAs that posttranscriptionally regulate protein expression. They are considered as essential regulators of development and physiological processes [18], and may play key roles in cancer progression via modulating oncogenic and tumor-suppressor pathways [19, 20]. Previous studies demonstrated several deregulated miRNAs may promote breast cancer proliferation and migration, including miR-340, miR-195 and miR-124 [21–23]. Recently, it was reported that miR-25, an MCU-targeting miRNA, reduces MCU expression and [Ca^2+^]_m_ uptake, thereby inducing resistance to apoptotic stimuli in and leading to increased survival of prostate and colon cancer cells [24]. Interestingly, miRNAs are also important to the metabolic shift in cancer cells [25, 26]. However, whether MCU-targeting miRNAs inhibit glycolysis and motility of breast cancer cells remains unclear.

The purpose of this study was to determine whether MCU promotes breast cancer metastasis through enhancing glycolysis, and whether this process is regulated by specific miRNAs. In the present study, we provide the first evidence that upregulated MCU expression enhances the metastatic capacity of breast cancer cells by inducing a shift from oxidative to glycolytic metabolism. We further demonstrate that miR-340 directly targets and downregulates MCU expression to suppress glycolysis and motility in breast cancer cells. Our results elucidate a novel mechanism, the miR-340/MCU pathway, is involved in breast cancer metastasis. This pathway may serve as a target for detecting, monitoring, and treating metastatic breast cancers.

## RESULTS

### MCU promotes breast cancer cell migration and invasion

**Figure 1:**
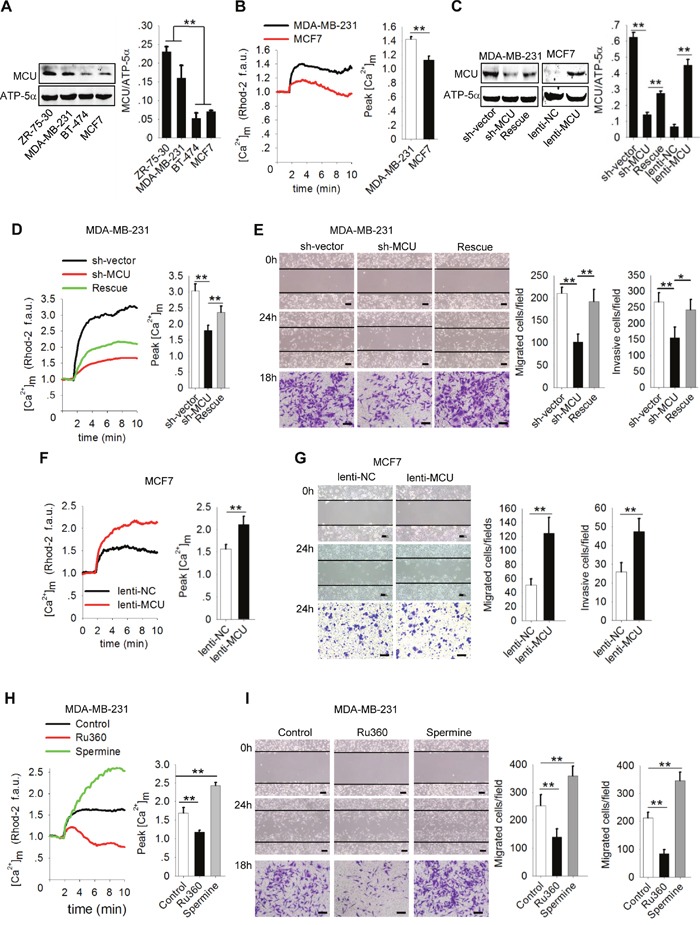
MCU promotes breast cancer cell migration and invasion **(A)** Western blots showing MCU protein expression levels in a panel of four breast cancer cell lines. **(B)** Kinetics of [Ca^2+^]_m_ in MDA-MB-231 and MCF7 cells according to Rhod-2 fluorescence imaging and quantification of [Ca^2+^]_m_ peak amplitudes after stimulation with 2mM CaCl_2_ (f.a.u., fluorescence arbitrary units). **(C)** Western blots showing MCU protein expression levels in MDA-MB-231 cells transfected with a lentivirus expressing sh-MCU, sh-NC or MCU sponge and in MCF7 cells transfected with lenti-MCU or lenti-NC. The blots shown are representative blots of MCU and ATP-5α from three independent experiments. **(D)** Kinetics of [Ca^2+^]_m_ in MDA-MB-231 cells transfected with a lentivirus expressing sh-MCU, sh-NC or MCU sponge according to Rhod-2 fluorescence imaging. **(E)** Wound-healing (top, middle) and Transwell invasion (bottom) assays of MDA-MB-231 cells transfected with a lentivirus expressing sh-MCU, sh-NC or MCU sponge. **(F)** Kinetics of [Ca^2+^]_m_ in MCF7 cells transfected with lenti-MCU or lenti-NC. **(G)** Wound-healing and Transwell invasion assays of MCF7 cells transfected with lenti-MCU or lenti-NC. **(H)** Kinetics of [Ca^2+^]_m_ in MDA-MB-231 cells treated with Ru360 (an MCU inhibitor) and spermine (an MCU agonist) according to Rhod-2 fluorescence imaging. **(I)** Wound-healing and Transwell invasion assays of MDA-MB-231 cells treated with Ru360, spermine, or control. Scale bars: 100μm. The error bars in all the bar graphs represent standard deviation (SD). Statistical significance was determined via one-way analysis of variance followed by pairwise *t*-tests.^*^P<0.05;^**^P<0.01.

It was recently reported that [Ca^2+^]_m_ homeostasis prompts sustained mROS production and contributes to tumor growth and metastasis [17]. To confirm that MCU, a major Ca^2+^ channel in the inner mitochondrial membrane, is involved in breast cancer cell migration and invasion, we measured MCU expression in four breast cancer cell lines with different metastatic potential (BT-474, MCF7, ZR-75-30, and MDA-MB-231). We found that MCU expression levels were significantly higher in ZR-75-30 and MDA-MB-231 cells, which are highly migratory and invasive, than in BT-474 and MCF7 cells, which exhibited poorer motility (P<0.01) (Figure 1A). In addition, the levels of [Ca^2+^]_m_ uptake were significantly higher in MDA-MB-231 cells than in MCF7 cells (Figure 1B). These data suggested that high MCU expression and associated high levels of [Ca^2+^]_m_ uptake promote breast cancer cell migration and invasion.

To confirm this finding, we transfected MDA-MB-231 cells with sh-MCU to knockdown the expression of MCU and then were restored the expression of MCU by transfecting MCU sponge, and infected MCF7 cells with lenti-MCU or lenti-NC. We confirmed knockdown, restoration and upregulation of MCU expression using Western blotting (Figure 1C). Knockdown of MCU expression in MDA-MB-231 cells produced a marked decrease in [Ca^2+^]_m_ uptake and in cell migration and invasion (Figure 1D, 1E). In contrast, restoration of the MCU expression in MDA-MB-231 cells or overexpression of MCU in MCF7cells significantly enhanced [Ca^2+^]_m_ uptake and cell migration and invasion (P<0.01) (Figure 1D-1G). Next, we used an MCU inhibitor, Ru360, to decrease [Ca^2+^]_m_ uptake and an MCU agonist, spermine, to increase [Ca^2+^]_m_ uptake (Figure 1H). Both migration and invasion of MDA-MB-231 cells were significantly inhibited by Ru360 and enhanced by spermine (Figure 1I). From these gain- and loss-of-function assays, we concluded that MCU promotes migration and invasion of breast cancer cells by regulating [Ca^2+^]_m_ uptake.

### MCU expression is associated with the Warburg effect in breast cancer cells

**Figure 2:**
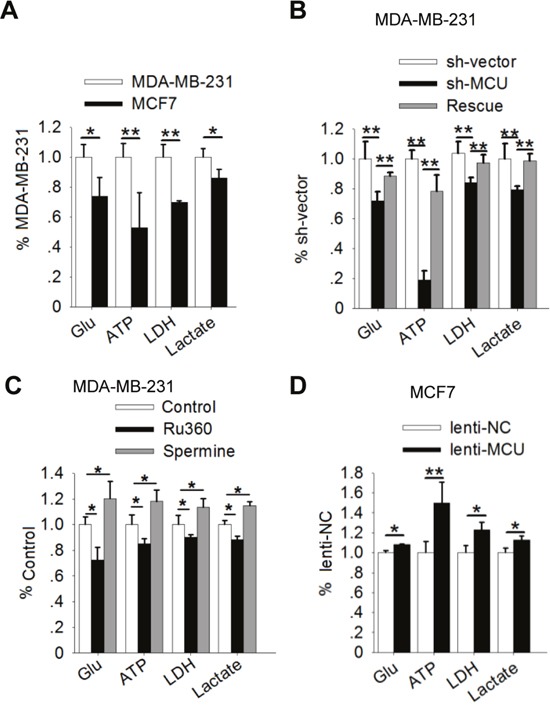
MCU expression promotes the Warburg effect in breast cancer cells **(A)** Glucose (Glu) uptake, ATP levels, LDH levels, and lactate production in MDA-MB-231 and MCF7 cells. **(B)** Glucose uptake, ATP levels, LDH levels, and lactate production in MDA-MB-231 cells transfected with a lentivirus expressing sh-MCU or sh-NC. **(C)** Glucose uptake, ATP levels, LDH levels, and lactate production in MDA-MB-231 cells treated with Ru360 (an MCU inhibitor) or spermine (an MCU agonist). **(D)** Glucose uptake, ATP levels, LDH levels, and lactate production in MCF7 cells transfected with lenti-MCU or lenti-NC. The error bars in all the bar graphs represent SD. Statistical significance was determined via one-way analysis of variance followed by pairwise *t*-tests.^*^P<0.05; ^**^P< 0.01.

Most cancer cells exhibit enhanced glycolysis: elevated glucose uptake and lactate production regardless of oxygen availability. This metabolic shift plays a role in enhancing cancer cell motility [5, 36, 37]. In our study, a comparison of the levels of glycolysis markers in breast cancer cells with different metastatic potentials demonstrated that glucose uptake, ATP levels, LDH levels, and lactate production were higher in the highly metastatic MDA-MB-231 cells than in the less metastatic MCF7 cells (Figure 2A).

Recently, investigators examined the role of mitochondria in regulating the metabolic alterations in cancer cells [6]. Because MCU is a key ion channel protein in the mitochondria [14], we assessed whether gain- and loss-of-function of MCU affects glycolysis in breast cancer cells. In both MCU-knockdown and Ru360-treated MDA-MB-231 cells, loss of MCU function was associated with significantly lower glucose uptake, ATP production, LDH levels, and lactate production than in control cells (Figure 2B, 2C). In contrast, restoration of the MCU expression in MDA-MB-231 cells, upregulation of MCU expression in MCF7 cells and activation of MCU function by spermine in MDA-MB-231 cells increased glucose uptake, ATP levels, LDH levels, and lactate production (Figure 2B-2D). These results suggested that upregulation of MCU expression promotes the Warburg effect in breast cancer cells.

### MCU is a downstream target of miR-340

**Figure 3:**
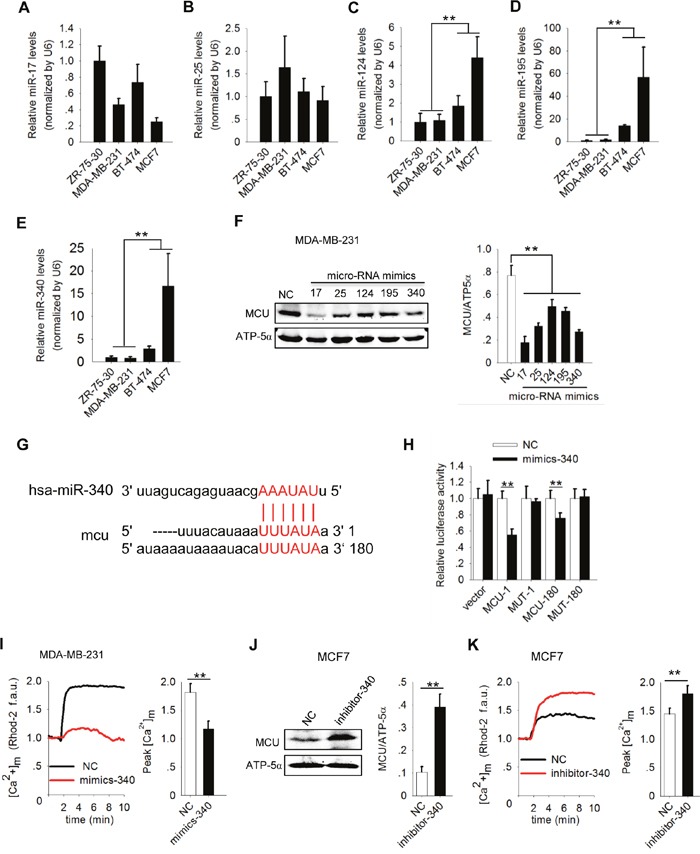
MiR-340 reduces [Ca2+]m in breast cancer cells by targeting MCU **(A-E)** Expression of miR-17, miR-25, miR-124, miR-195 and miR-340 in a panel of four breast cancer cell lines by quantitative polymerase chain reaction analysis. **(F)** Western blots showing MCU protein expression in MDA-MB-231 cells expressing five different miRNAs. **(G)** Sequences present in the 3′ -UTR of MCU targeted by miR-340 and its target region are highlighted. **(H)** Luciferase assay showing the effect of miR-340 expression on luciferase reporter gene activity when linked with the targeted segment of the 3′ -UTR of MCU in MDA-MB-231 cells. **(I)** Kinetics of [Ca^2+^]_m_ in MDA-MB-231 cells expressing miR-340 (mimics-340) and NC cells according to Rhod-2 fluorescence imaging (f.a.u., fluorescence arbitrary units). **(J)** Western blot showing MCU protein expression levels in MCF7 cells expressing a miR-340 inhibitor (inhibitor-340). **(K)** Kinetics of [Ca^2+^]_m_ in MCF7 cells expressing inhibitor-340 according to Rhod-2 fluorescence imaging. The error bars in all the bar graphs represent SD. Statistical significance was determined via one-way analysis of variance followed by pairwise *t*-tests.^*^P<0.05; ^**^P< 0.01.

Since specific miRNAs are up- or downregulated, with consequent alteration in the expression of target proteins, we screened the output of three target prediction algorithms (MicroRNA, starBase, and TargetScan; Table 1) to determine whether miRNAs regulate MCU expression. The screening predicted five breast cancer-related miRNAs (miR-17, miR-25, miR-124, miR-195, and miR-340) to target MCU. We detected endogenous expression of all five miRNAs in breast cancer cell lines. There are significant differences between high and low metastatic breast cancer cell lines in the expression levels of miR-124, miR-195 or miR-340 (Figure 3A-3E). In MDA-MB-231 cells, overexpression of all five identified miRNAs reduced MCU expression; the lowest expression levels were associated with overexpression of miR-17 and miR-340(Figure 3F). We therefore selected miR-340 for further study.

**Table 1:** Effect of five miRNAs targeting MCU mRNA in human breast cancers

Algorithm	miRNA	Breast cancer	Glucose metabolism	MCU
MicroRNA	miR-340	Down	Down	No report
starBase	miR-124	Down	Down	No report
TargetScan	miR-25	Down	Down	Down
	miR-195	No report	Down	No report
	miR-17	No report	Down	No report

miRNA, microRNA; MCU, mitochondrial calcium uniporter.

To obtain additional direct evidence that MCU expression is inhibited by miR-340, we identified the binding sites for miR-340 in the 3 ′ -UTR of MCU at two regions and created luciferase reporters to assess the direct interactions of miR-340 and MCU mRNA in MDA-MB-231 cells (Figure 3G). We co-transfected two MCU 3 ′ -UTR reporters (MCU-1 and MCU-180), corresponding mutant reporters (MUT-1 and MUT-180) with mimics-340, or NC into MDA-MB-231 cells. The luciferase activity in both MCU-1 and MCU-180 was lower in the presence of mimics-340 than in that of NC. We observed no significant change in luciferase activity for either MUT-1 or MUT-180 in the presence of mimics-340 (Figure 3H). Our data suggested that MCU mRNA is a direct physical target of miR-340.

To further validate the effects of miR-340 on the function of MCU as a Ca^2+^ channel, we measured [Ca^2+^]_m_ uptake in MDA-MB-231 cells expressing miR-340. [Ca^2+^]_m_ uptake was markedly lower in mimics-340–expressing cells than in NC cells (*P*<0.01) (Figure 3I). In contrast, inhibiting miR-340 expression in MCF7 cells with inhibitor-340 increased MCU expression (Figure 3J) and [Ca^2+^]_m_ uptake(Figure 3K). Taken together, these gain- and loss-of-function experiments further demonstrated that miR-340 targets MCU and reduces MCU expression, resulting in reduced [Ca^2+^]_m_ uptake in breast cancer cells with different metastatic potentials.

### MiR-340 inhibits motility and the Warburg effect in breast cancer cells

**Figure 4:**
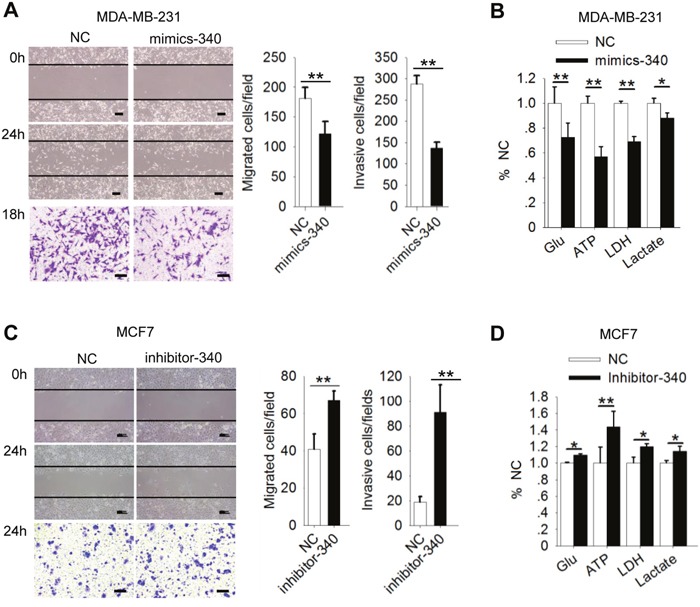
MiR-340 inhibits motility of and the Warburg effect in breast cancer cells **(A)** Wound-healing (top, middle) and Transwell invasion (bottom) assays of MDA-MB-231 cells treated with an miR-340 mimic (mimics-340) or NC. **(B)** Glucose (Glu) uptake, ATP levels, LDH levels, and lactate production in MDA-MB-231 cells expressing mimics-340. **(C)** Wound-healing and Transwell invasion assays of MCF7 cells expressing inhibitor-340. **(D)** Glucose uptake, ATP levels, LDH levels, and lactate production in MCF7 cells expressing inhibitor-340. The error bars in all the bar graphs represent SD. Statistical significance was determined via one-way analysis of variance followed by pairwise *t*-tests.^*^P<0.05; ^**^P< 0.01.

As described above, MCU overexpression promoted motility and enhanced the Warburg effect in breast cancer cells, and miR-340 directly targeted MCU mRNA to suppress MCU expression in these cells. We therefore investigated the role of miR-340 in regulating cell motility and the Warburg effect in breast cancer cells with different metastatic potentials. MDA-MB-231 cells treated with mimics-340 were significantly less migratory and invasive than the cells treated with NC (Figure 4A). Consistent with the findings shown in Figures 1-3, treatment with mimics-340 significantly decreased glucose uptake, ATP levels, LDH levels, and lactate production in highly metastatic MDA-MB-231 cells (Figure 4B). In contrast, inhibition of miR-340 increased migration and invasion of less metastatic MCF7 cells (Figure 4C) and enhanced glucose uptake, ATP levels, LDH levels, and lactate production in these cells (Figure 4D). These results demonstrated that miR-340 expression regulates glycolysis and MCU expression, leading to reduced [Ca^2+^]_m_ uptake and motility in breast cancer cells.

### MiR-340 inhibits cell motility via directly targets MCU

**Figure 5:**
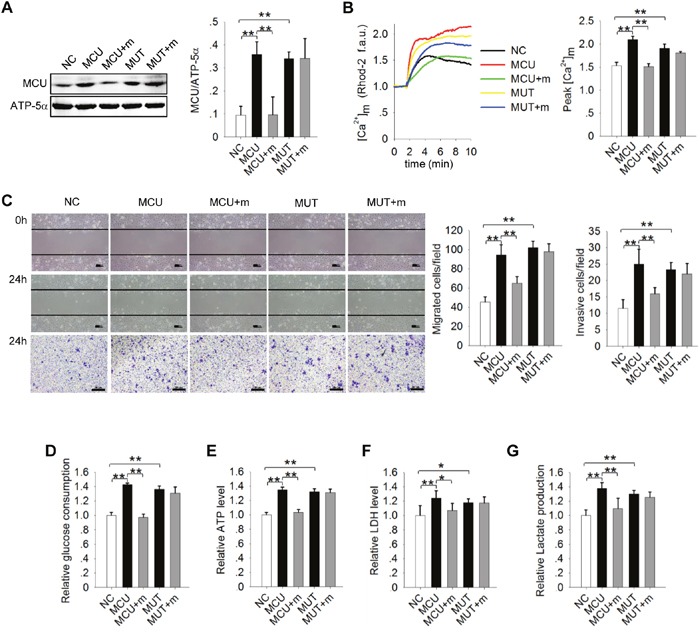
Direct targeting of MCU by miR-340 inhibits motility of and the Warburg effect in MCF7 cells **(A)** Western blots showing MCU expression levels in MCF7 cells. MiR-340 was co-transfected with an MCU–wild-type construct (MCU), an MCU mutant construct in which the target sequence of miR-340 was mutated (MUT), or NC. ATP-5α was used as an internal control. **(B)** Kinetics of [Ca^2+^]_m_ in MCF7 cells treated as in (A) according to Rhod-2 fluorescence imaging. **(C)** Wound-healing (top, middle) and Transwell invasion (bottom) assays in MCF7 cells treated as in (A). **(D–G)** Glucose uptake, ATP levels, LDH levels, and lactate production in MCF7 cells treated as in (A). The error bars in all the bar graphs represent SD. Statistical significance was determined via one-way analysis of variance followed by pairwise *t*-tests.^*^P<0.05; ^**^P< 0.01.

Previous research demonstrated that miR-340 inhibits cell migration and invasion by targeting multiple negative regulators of the c-Met and Pumilio proteins [38, 39]. To determine whether miR-340 also directly targets MCU to inhibit cell migration and invasion, we transfected a pcDNA vector carrying MCU or MUT into MCF7 or MDA-MB-231 cells, separately. Transfection with either pcDNA-MCU or pcDNA-MUT increased MCU protein expression, whereas transfection with pcDNA-MCU did not increase MCU expression in the presence of mimics-340(Figure 5A). Consistently, the MCU expression in MDA-MB-231 cells with miR-340 overexpressing was decreased, however, pcDNA-MUT rescued the MCU expression (Supplementary Figure 1A). Assessment of [Ca^2+^]_m_ uptake (Figure 5B and Supplementary Figure 1B), migration and invasion (Figure 5C and Supplementary Figure 1C), glucose uptake, ATP levels, LDH levels, and lactate production (Figure 5D-5G and Supplementary Figure 1D) confirmed that miR-340 directly targets MCU to inhibit both cancer cell migration and invasion and glycolysis.

### MCU and miR-340 expression affect breast cancer metastasis *in vivo*

**Figure 6:**
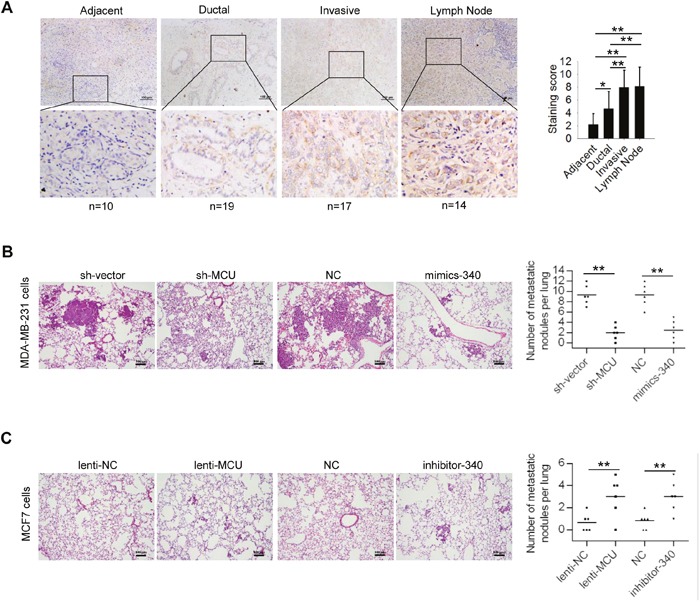
MCU and miR-340 expression regulate breast cancer metastasis *in vivo* **(A)** (Left) Immunohistochemical stains of representative samples of human breast carcinoma sections showing MCU expression. Scale bars (top row): 100 μm; inset (bottom row) scale: 50 μm. (Right) Immunohistochemical staining scores for the tissue samples examined: tissue adjacent to the tumor (n=5), ductal cancer tissue (n=14), invasive cancer tissue (n=12), and lymph node tissue (n=9). **(B)** Hematoxylin and eosin staining of representative lung tissue sections from mice injected with MDA-MB-231 harboring sh-MCU, miR-340, or NC. **(C)** Hematoxylin and eosin staining of representative lung tissue sections from mice injected with MCF7 breast cancer cells harboring lenti-MCU, inhibitor-340, or NC. Scale bars: 100μm. The number of lung metastatic foci in each group was determined in five randomly selected fields. The error bars in all the bar graphs represent SD. Statistical significance was determined via one-way analysis of variance followed by pairwise *t*-tests.^*^P< 0.05; ^**^P< 0.01.

To determine whether our findings are clinically relevant, we examined the expression levels of MCU in breast tumor samples collected from 60 patients with different clinical and pathological characteristics (Table 2). We found that MCU expression levels were low in normal tumor-adjacent tissue and in ductal carcinoma tissue. In contrast, we observed high MCU expression in tumor samples from patients with distant metastasis and those whose cancer had spread to the lymph nodes (Figure 6A).

**Table 2:** Clinical and pathological characteristics of 60 patients with breast cancer

Characteristic	N	Tissue type				*P* value
		Adjacent	Ductal carcinoma	Invasive	Lymph node	
Number of samples	60	10	19	17	14	-
Patient age in years (mean ± SD)	56.72±7.14	54.30±6.04	52.68±7.30	60.59±6.06	59.21±5.71	P=0.002
Tumor stage						
T1, T2	42	10	19	7	6	*P*<0.001
T3, T4	18	0	0	10	8	
Lymph node metastasis						
No	29	10	19	0	0	*P*<0.001
Yes	31	0	0	17	14	
Distant metastasis						
No	45	10	19	9	7	*P*<0.001
Yes	15	0	0	8	7	
Stage group						
I, II	40	10	19	6	5	*P*<0.001
III, IV	20	0	0	11	9	
ER+						
No	25	6	7	6	6	P=0.529
Yes	35	4	12	11	8	

SD, standard deviation; ER, estrogen receptor.

Furthermore, to ascertain the relationship of MCU and miR-340 expression with breast cancer metastasis *in vivo*, we generated an experimental lung metastasis model in nude mice. We injected mice via the tail vein with MDA-MB-231 cells with downregulated MCU expression, MDA-MB-231 cells with overexpressed miR-340, MCF7 cells with overexpressed MCU, or MCF7 cells with downregulated miR-340 expression. We killed the mice 42 days after injection (n=6 in each group). We found fewer metastatic lung nodules in the mice that had been injected with MCU-downregulated or miR-340–overexpressing MDA-MB-231 cells than in the control group (Figure 6B). In contrast, we found more metastatic lesions in the lungs of mice injected with MCU-overexpressing or miR-340–downregulated MCF7 cells than in the control group (Figure 6C). These results demonstrated that MCU facilitates metastasis of breast cancer cells *in vivo* and that miR-340 silences MCU and inhibits the development of metastatic lesions in the lungs.

## DISCUSSION

In this study, we demonstrated that MCU drives metastasis through the upregulation of glycolysis in breast cancer cells. We also found that miR-340 decreases glycolysis and motility of breast cancer cells likely via direct suppression of MCU expression by posttranscriptional downregulation.

Recently, the roles of [Ca^2+^]_m_ uptake in tumor growth and metastatic formation was intensively investigated [17, 40]. The molecular characterization of MCU confirmed that it is a selective channel responsible for [Ca^2+^]_m_ uptake. These studies provided the basis for investigating the explicit role of [Ca^2+^]_m_ uptake under several pathophysiological conditions, including cancer [14, 15]. Moreover, several previous studies demonstrated that MCU is highly expressed in estrogen receptor-negative and basal-like breast cancers, both of which have poor prognoses [16, 41, 42]. Similarly, we demonstrated that MCU expression is positively associated with metastasis in human breast cancer cell lines. Tosatto *et al.* [17] also showed that MCU suppression strongly reduces [Ca^2+^]_m_ uptake in and migration of breast cancer cells. Taken together, findings from our studies and others provide new evidence of MCU involvement in driving metastatic progression in breast cancer.

Like Su *et al.* [37], we found higher expression of glycolysis marker, [Ca^2+^]_m_ uptake, and higher MCU expression in MDA-MB-231 cells than in MCF7 cells. We thus speculated that elevated [Ca^2+^]_m_ uptake is essential for the metabolic shift that occurs during cancer metastasis. To validate our hypothesis, we markedly reduced [Ca^2+^]_m_ uptake and found that glycolysis was strongly inhibited. Conversely, activation or overexpression of MCU increased glycolysis and metastasis. These results point to a role for MCU in regulation of the metabolic shift from oxidative phosphorylation to aerobic glycolysis. However, identifying the mechanism by which MCU is implicated in the metabolic shift during cancer progression requires further investigation. MCU may be related to elevated [Ca^2+^]_m_ uptake, which may induce the destruction of the mitochondrial respiratory chain [6], or to elevated cytoplasmic calcium levels induced by store-operated Ca^2+^entry channels that are regulated by MCU-enhanced glycolysis [12].

The miRNAs are known to regulate the translation of distinct target transcripts and have been implicated in the development and spread of cancer [18, 19]. To determine whether posttranscriptional regulation by certain miRNAs is an upstream regulatory mechanism of MCU expression, we used target-prediction algorithms to determine miRNA binding sites in the MCU 3′-UTR [24]. We identified miR-17 and miR-340 binding sites but found that only miR-340expression was inversely correlated with the metastatic potential of breast cancer cells. Notably, when miR-340 was expressed in highly metastatic MDA-MB-231 cells, MCU expression and function were significantly suppressed, and [Ca^2+^]_m_ uptake was significantly reduced. Additionally, increased MCU expression and [Ca^2+^]_m_ uptake in miR-340–downregulatedMCF7 cells further confirmed that miR-340 negatively regulates MCU expression. Taken together, these results illustrated that miR-340 suppressed metastasis and glycolysis via targeting MCU. MiR-340 has been shown to regulate other targets involved in cancer cell migration and invasion. A previous study demonstrated that miR-340 reverses cisplatin resistance in hepatocellular carcinoma cell lines by targeting the nuclear factor (erythroid-derived 2)-like 2-dependent antioxidant pathway [43]. MiR-340 was also reported to be involved in suppressing migration and invasion by targeting MYO10 [44] or c-Met [38] in breast cancer cells. Our results demonstrated that miR-340 inhibited metastasis of breast cancer cells by targeting MCU and reversing the metabolic shift to aerobic glycolysis. Whether MCU expression is connected with MYO10 or c-Met in breast cancer remains unraveled.

In conclusion, our study demonstrated that MCU-mediated [Ca^2+^]_m_ uptake is required for breast cancer metastatic progression and that MCU-mediated [Ca^2+^]_m_ uptake is closely correlated with the Warburg effect. It also identified a new MCU-regulatory pathway involving miR-340, a miRNA associated with the Warburg effect. Our discovery of this pathway reveals new opportunities to prevent cancer from metastasizing and suggests that blockade of MCU expression is a novel strategy for treating subtypes of breast cancer with elevated MCU levels and enhanced glycolysis. Further exploration of the mechanisms by which MCU enhanced the Warburg effect in breast cancer cells is warranted.

## MATERIALS AND METHODS

### Cell culture

The human breast carcinoma cell lines ZR-75-30, MDA-MB-231, MCF7, and BT-474 were purchased from ATCC (Manassas, VA). Briefly, the cells were cultured in Dulbecco's modified Eagle's medium (DMEM) or RPMI-1640 medium supplemented with 10% fetal bovine serum (FBS) (Life Technologies/Invitrogen, Carlsbad, CA) in 5% CO_2_ at 37°C. Short tandem repeat (STR) profiling was used to validate breast cancer cell lines. All experiments were performed in passage 3 cells.

### Lentivirus production and vector constructs

A miR-340 mimic (mimics-340), a miR-340 inhibitor (inhibitor-340), and negative control (NC) oligonucleotides were purchased from RiboBio (Guangzhou, People's Republic of China) and transfected into cells using Lipofectamine 2000 (Invitrogen, U.S.A.) at a concentration of 50 nM. Lentiviral plasmids containing MCU, a MCU-specific short hairpin RNA (sh-MCU), and NC were purchased from Cyagen (Shanghai, China). Lentiviral plasmids expressing mimics-340, inhibitor-340, and NC were purchased from Obio Technology (Shanghai, China). A MCU-expressing vector with miR-340–binding sites (MCU-1 and MCU-180) or mutated seed sequences of miR-340 at the MCU 3′-untranslated region (UTR; MUT-1 and MUT-180) was purchased from GeneChem (Shanghai, China) and was subcloned into the psiCHECK-2 vector, followed by transfecting into cells using Lipofectamine 2000 (Invitrogen, U.S.A.).

### Western blot analysis

Western blot studies were performed as described previously [27]. The primary antibodies used were those against MCU and ATP-5α (Abcam, Cambridge, MA). IRDye800 and IRDye680 (LI-COR Biosciences, Lincoln, NE) were used as secondary antibodies. Signal intensities for MCU and ATP-5α were analyzed using an Odyssey Infrared Image System (LI-COR Biosciences).

### Measurement of [Ca^2+^]_m_ concentrations in breast cancer cells

Either MDA-MB-231 cells or MCF7 cells grown on glass-bottomed petri dishes were loaded with 2μM Rhod-2/AM for 30min in Hank's balanced salt solution (Gibco BRL, San Diego, CA). After 100s of baseline image recording, CaCl_2_ was added to the petri dish to the final concentration of 2mM, and confocal images were recorded for 10min by an inverted microscope (FV1000-IX71, Olympus, Tokyo, Japan) at 488-nm and 561-nm excitation using a 20×objectivelens [28].

### Invasion assays

All invasion assays were performed as described previously [29]. A total of 5×10^4^ MDA-MB-231 or MCF7 cells were placed in the top compartment of a Transwell chamber (Millipore, U.S.A.). Later, the membranes were stained with crystal violet and photographed using an inverted microscope (IX51, Olympus, Tokyo, Japan) at a 20×objective lens.

### Wound-healing assays

To determine their capacity for migration, wound-healing assays were performed as described previously [17]. Wounds were made by sterile pipette tips. At 24h, the cells in the denuded zone of each dish were counted and photographed at 20×magnification using an inverted microscope (IX51, Olympus, Tokyo, Japan) in a random fashion.

### Adenosine triphosphatelevels

Adenosine triphosphate (ATP) levels of cells were determined using an ATP testkit according to the manufacturer's instructions (Beyotime Institute of Biotechnology, Jiangsu, China) as previously described [30].

### Analysis of lactate dehydrogenase activation, glucose uptake, and lactate production

Lactate dehydrogenase (LDH) activities of MDA-MB-231 (NC short hairpin RNA [sh-vector], sh-MCU, NC, and mimics-340) and MCF7 (lenti-NC, lenti-MCU, NC, and inhibitor-340) cells were determined with an LDH assay kit according to the manufacturer's instructions (Jiancheng Bioengineering Institute, Nanjing, China). Glucose uptake levels were determined by measuring the uptake of ^3^H-2-deoxyglucose as described previously [31]. Lactate production levels in the culture medium were measured using a lactate assay kit (Jiancheng Bioengineering Institute).

### RNA extraction and miRNAs analysis

Total RNA was extracted from ZR-75-30, MDA-MB-231, MCF7, and BT-474 cells using the Trizol reagent (Invitrogen, Carlsbad, CA). The miRNA levels were quantified by qRT-PCR using TaqMan assay kits (ABI) with U6 snRNA as the reference, as previously described [32, 33].

### Luciferase assays

The 3′-UTRs of MCU-1, MCU-180, the mutated 3′-UTRs MCU-1 (MUT-1) and MCU-180 (MUT-180) were amplified and inserted downstream from the stop codon of Renilla luciferase using the psiCHECK-2 vector (Sagene). MDA-MB-231 cells were cultured in 96-well plates and co-transfected with 10 ng psiCHECK-2-MCU-1/180 3′-UTR or psiCHECK-2-MUT-1/180 3′-UTR plasmid and 5pmol mimics-340 or NC. After 48 h of incubation, firefly and Renilla luciferase activities were measured using a Dual-Luciferase Reporter Assay System (Promega, Madison, WI) as described previously [25].

### Human breast cancer tumor samples, immunohistochemistry, and scoring

The Institutional Human Subject Research Review Committee of the Southern Medical University approved all procedures performed in human subjects. Sixty patients provided informed consent and had undergone radical resection of their breast tumors and lymph node dissection at the Southern Medical University Hospital from June 2011 to December 2012. Tumors had been confirmed histopathologically and staged according to the Union for International Cancer Control TNM system at the time of resection [34]. The records were collected in the department of case of Southern Hospital. Paraffin-embedded sections of the tumor samples and samples of tissues adjacent to the tumors were collected from the Pathology Department of Southern Medical University Hospital and subjected to immunohistochemical staining with an anti-MCU antibody (Abcam). The MCU expression levels in the tumor samples and samples of tissues adjacent to the tumors were scored according to the percentage of MCU-positive cells in each area of breast cancer tissue and the staining intensity of the MCU-positive cells as described previously [35].

### Mouse models of breast cancer and lung metastasis

Animal studies were performed according to protocols approved by the Institutional Animal Care and Use Committee of the Standing Committee on Animals at the Southern Medical University. Female NOD/SCID mice (15–20 g, 8–10 weeks of age) (Laboratory Animal Center, Southern Medical University, Guangzhou, China) were housed in cages with high-efficiency particle-arrest–filtered air and housed using a12-h light-dark cycle. The mice were provided *ad libitum* access to food and autoclaved water. The mice were placed randomly into eight groups according to the type of cell injected into them (n=6 per group): MDA-MB-231 (NC short hairpin RNA [sh-vector], sh-MCU, NC, and mimics-340) and MCF7 (lenti-NC, lenti-MCU, NC, and inhibitor-340). Cells (2×10^6^) were introduced via injection into the tail vein. After 6 weeks, the mice were sacrificed by neck breaking and their lungs were removed, mounted on the slides for hematoxylin and eosin staining, and counted the metastatic nodules for analyzing the macrometastatic and micrometastatic lesions.

### Statistical analysis

All of cell lines experiments were carried out in a minimum of three replications. All quantified xenograft and *in vitro* assay results are presented as means ± standard deviations. Statistical analyses were conducted using the SPSS version 13.0 software program (SPSS Institute, Chicago, IL). All statistical tests were two-sided. Student *t*-tests were used to compare continuous variables. Statistical significance was defined as P<0.05 or P<0.01.

## SUPPLEMENTARY MATERIALS FIGURE



## Abbreviations

MCU: mitochondrial calcium uniporter
[Ca^2+^]_m_: mitochondrial Ca^2+^
miRNA: microRNA
ATP: adenosine triphosphate
NC: negative control
DMEM: Dulbecco's modified Eagle's medium
FBS: fetal bovine serum
STR: Short tandem repeat
shRNA: short hairpin RNA
UTR: untranslated region
LDH: lactate dehydrogenase
f.a.u.: fluorescence arbitrary units
