# Supplementary material for: Mitochondrial calcium uniporter as a target of microRNA-340 and promoter of metastasis via enhancing the Warburg effect
Source: Oncotarget. 2017 Jul 31;8(48):83831–44. doi: 10.18632/oncotarget.19747 (PMC5663558; doi:10.18632/oncotarget.19747)
Supplement: Supplementary file 1 [file oncotarget-08-83831-s001.pdf]

Mitochondrial calcium uniporter as a target of microRNA-340 and promoter of metastasis via enhancing the Warburg effect

SUPPLEMENTARY MATERIALS

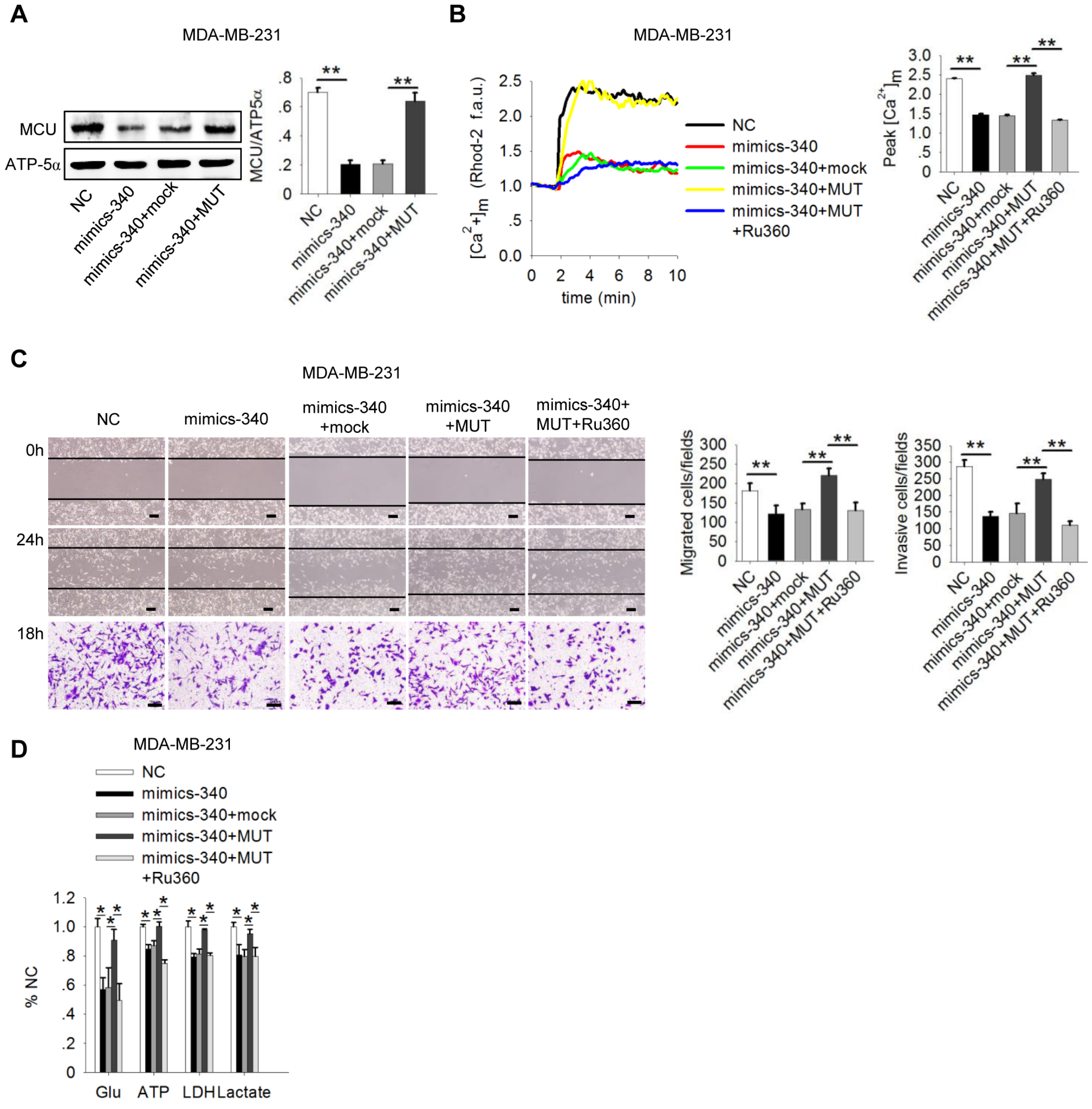

Supplementary Figure 1: Direct targeting of MCU by miR-340 inhibits motility of and the Warburg effect in MDA-MB-231 cells. (A) Western blots showing MCU expression levels in MDA-MB-231 cells. MiR-340 was transfected or co-transfected with an MCU mutant construct in which the target sequence of miR-340 was mutated (MUT), or mock. ATP-5α was used as an internal control. **(B)** Kinetics of [Ca2+]m in MDA-MB-231 cells treated as in (A) and Ru360 pretreated (10μM, 10min) according to Rhod-2 fluorescence imaging. **(C)** Wound-healing (top, middle) and Transwell invasion (bottom) assays in MDA-MB-231 cells treated as in (B). **(D)** Glucose uptake, ATP levels, LDH levels, and lactate production in MDA-MB-231 cells treated as in (B). Scale bars: 100μm. The error bars in all the bar graphs represent SD. Statistical significance was determined via one-way analysis of variance followed by pairwise t-tests. *P<0.05; **P< 0.01.
